# LipidII: Just Another Brick in the Wall?

**DOI:** 10.1371/journal.ppat.1005213

**Published:** 2015-12-17

**Authors:** Dirk-Jan Scheffers, Menno B. Tol

**Affiliations:** Department of Molecular Microbiology, Groningen Biomolecular Sciences and Biotechnology Institute, University of Groningen, The Netherlands; Stony Brook University, UNITED STATES

## Abstract

Nearly all bacteria contain a peptidoglycan cell wall. The peptidoglycan precursor molecule is LipidII, containing the basic peptidoglycan building block attached to a lipid. Although the suitability of LipidII as an antibacterial target has long been recognized, progress on elucidating the role(s) of LipidII in bacterial cell biology has been slow. The focus of this review is on exciting new developments, both with respect to antibacterials targeting LipidII as well as the emerging role of LipidII in organizing the membrane and cell wall synthesis. It appears that on both sides of the membrane, LipidII plays crucial roles in organizing cytoskeletal proteins and peptidoglycan synthesis machineries. Finally, the recent discovery of no less than three different categories of LipidII flippases will be discussed.

Peptidoglycan (PG), the main component of the cell wall, is a structure unique to bacteria. Currently, over 50% of the antibiotics in use target bacterial cell wall synthesis, and thus PG synthesis is considered the Achilles’ heel of bacteria [1]. The precursor of PG is LipidII, a lipid-linked disaccharide with a pentapeptide side chain. Linkage of the disaccharide to a growing glycan strand results in release of the lipid anchor and leaves the pentapeptide free for crosslinking to peptides on other glycan strands or for processing. Various excellent reviews describe the synthesis of LipidII, the incorporation of LipidII into PG, and the use of LipidII as a target for antibacterials [2–6]. LipidII’s conserved structure makes it difficult for pathogens to develop resistance against LipidII targeting molecules. This review focuses on the latest findings on antibacterials targeting LipidII, such as teixobactin [7], and on new LipidII biology (summarized in Fig 1). It is becoming more and more evident that LipidII is not just a passive brick that is being added to the cell wall but rather plays a key role in organization of the membrane.

**Fig 1:**
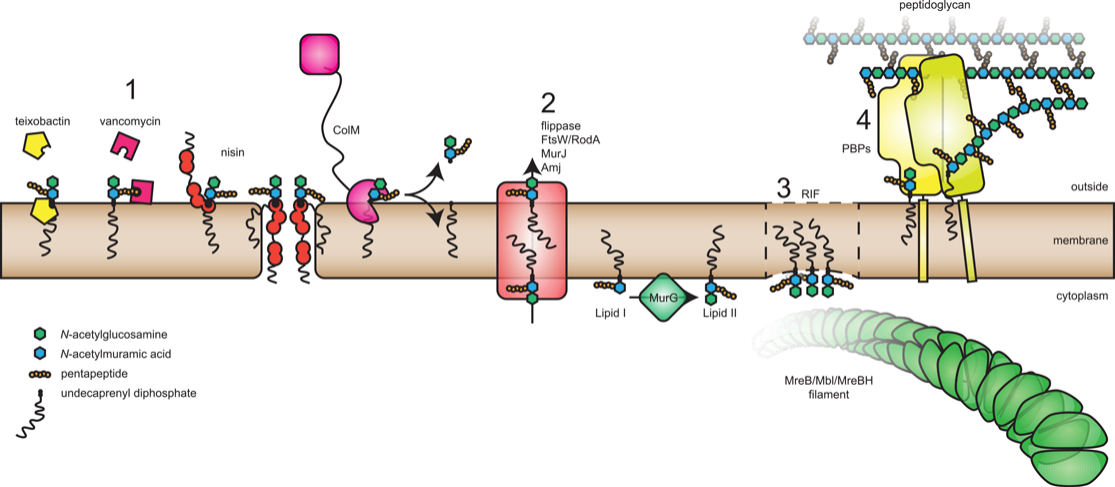
Organization of cell wall synthesis by LipidII. Overview of recent work that highlights various new insights about the role of LipidII; for example, (1) in the identification of novel antibacterials that target LipidII (including teixobactin and bacteriocins), (2) how LipidII is translocated over the membrane by different families of flippases (such as FtsW or RodA, MurJ, and Amj), (3) how it is recruited to regions of increased fluidity (RIFs) and how it organizes attachment of MreB(-like) filaments, and (4) how cell wall synthesis enzymes (penicillin-binding proteins [PBPs]) are recruited to LipidII.

## LipidII in Pathogens and As a Target for Antibacterials

Cell wall biosynthesis inhibitors are the most used antibacterial drugs worldwide, and the essentiality and uniqueness of the bacterial cell wall make it an ideal target for antibiotics [1]. LipidII is a particularly attractive target, as it is highly conserved and difficult to modify [1,8]. PG is even more ubiquitous than was thought. Recently, the Chlamydia anomaly—the observation that Chlamydia has no detectable PG but is sensitive to penicillin—was solved by novel fluorescent labeling methods that showed that these bacteria do indeed contain peptidoglycan [9,10]. That Chlamydiae (and Wolbachia) contain LipidII was already known [11], but LipidII was thought to be only required to organize cellular processes such as division in these organisms.

Various classes of compounds target LipidII (Fig 1), including (i) glycopeptides, like vancomycin, that bind the last two D-amino acid residues of the pentapeptide blocking crosslinking [12]—though hydrophobic derivatives of vancomycin also block transglycosylation [13]; (ii) unmodified peptides, like defensins, which are part of the innate immune system [14]; (iii) lantibiotics, such as nisin, which are peptides that contain thioether rings formed by posttranslational modification, [15]; and (iv) depsipeptides, like teixobactin, which are nonribosomally synthesized peptides [16]. In addition, various compounds inhibit the synthesis of LipidII (D-cycloserine, fosfomycin, tunicamycin) or the recycling of the undecaprenol carrier molecule, which results in depletion of LipidII (bacitracin) [1].

Recently, teixobactin, a LipidII-targeting antibiotic isolated from the gram-negative soil bacterium *Eleftheria terrae*, was discovered [7]. Teixobactin is a nonribosomally synthesized depsipeptide that binds the pyrophosphate-sugar moiety of undecaprenyl-bound cell wall precursors such as LipidI, II, and III (precursor for wall teichoic acid), thus preventing synthesis of the cell wall and killing the cells. Teixobactin is active against several gram-positive bacteria and kills pathogens, including *Mycobacterium tuberculosis*, *Staphylococcus aureus*, and various drug-resistant strains, possibly without development of resistance [7]. Unlike various other peptides that target LipidII, teixobactin is very stable and has low toxicity, making it one of the most promising antibacterial compounds discovered in the last three decades.

In the last few years, a number of papers have been published on the mechanism of lantibiotics, short peptides containing lanthionine and methyl-lanthionine rings [17]. Two classes are distinguished: Class A lantibiotics that have elongated structures and Class B lantibiotics with globular structures. Nisin is the most well-known Class A lantibiotic. Class A lantibiotics contain a “pyrophosphate cage” consisting of two lanthionine rings that bind LipidII [18], which results in insertion of the elongated peptide in the membrane and the formation of large, stable pores composed of four LipidII and eight nisin molecules [8]. Pore formation is sufficient to kill cells, but the binding to the LipidII pyrophosphate moiety prevents LipidII incorporation into glycan strands, a second killing mechanism [19]. The binding of nisin to LipidII also results in the formation of clusters of LipidII in the membrane, removing LipidII from the normal sites of PG synthesis [20]. Formation of these clusters is concomitant with pore formation, and these pores are sufficient to kill L-forms, cells that proliferate without an intact cell wall [21]. Class B lantibiotics, such as mersacidin, bind to LipidII but do not form membrane pores. The unavailability of LipidII for incorporation into PG and the sequestration of the undecaprenol carrier molecule are sufficient to kill the cell.

Several two-component lantibiotics were recently discovered, such as Lacticin 3147 [22] and haloduracin [23]. These lantibiotics act synergistically: both components are needed for killing. It is thought that the first component, which is structurally similar to mersacidin, binds to LipidII and triggers a conformational change that enables binding of the second component, which has a high similarity with the C-terminal tail of nisin, resulting in pore formation and cell death [24]. The hybrid lantibiotic microbisporicin (commercially developed as NAI-107) combines these two components in one molecule [25,26]. The C-terminal part of microbisporicin is similar to the C-terminal parts of nisin and gallidermin, while the N-terminal part resembles mersacidin. These newly discovered lantibiotics increase our understanding of the mechanism of known lantibiotics, and are also important candidates for clinical development.

Resistance against antibacterials that target LipidII does not develop easily. Vancomycin resistance occurs by replacement of the terminal D-Ala-D-Ala part of the pentapeptide with D-Ala-D-Lac and appeared only after 30 years of use in the clinic [27]. The pyrophosphate and the adjacent MurNAc sugar group of LipidII are highly conserved, and resistance development against antibacterials targeting this site of LipidII is expected to be even tougher. For instance, nisin has been used for more than four decades in the food industry without significant resistance development [15]. Also, various pathogens failed to develop resistance against the recently discovered teixobactin [7]. These classes of antibacterials are promising therapeutics, although teixobactin and lantibiotics only target LipidII in gram-positive bacteria, as they do not pass the outer membrane in gram-negatives.

## Flipping LipidII across the Cytoplasmic Membrane

LipidII synthesis in the cytoplasm and its incorporation into PG are well characterized, yet how LipidII is flipped across the membrane has long remained a mystery. Recent work by the Breukink, Ruiz, Bernhardt, and Rudner labs has identified three different protein classes that can translocate LipidII: FtsW (and its homologue RodA), MurJ, and Amj.

FtsW and RodA are members of the Shape, Elongation, Divison, and Sporulation (SEDS) protein family, which is conserved among PG-containing bacteria but absent from some wall-less bacteria or archaea [28]. The *ftsW* and *rodA* genes code for integral membrane proteins (IMP) that generally contain ten transmembrane α-helical segments (TMS), thus fitting a transport protein function, and are often organized in an operon structure with a cognate transpeptidase that is required for correct PG organization. The FtsW-transpeptidase combination is required for PG synthesis at the division site and depends on FtsZ for localization [29,30], whereas the RodA-transpeptidase combination functions in sidewall synthesis; the interaction between RodA and its cognate transpeptidase is dependent on active MreB [31,32] in bacteria in which MreB homologues are present. The Breukink lab synthesized a fluorescent LipidII analogue for use in in vitro flipping assays with membrane vesicles or proteoliposomes [33]. The amount of FtsW in membrane vesicles determined the amount of LipidII translocated, and reconstitution of purified FtsW in proteoliposomes showed that FtsW is sufficient to mediate LipidII translocation. Control proteins such as KcsA and SecYEG, as well as MurJ, did not transport LipidII [33]. Strikingly, TMS5–TMS10 are not required for in vitro LipidII transport, but two positively charged residues in TMS4 are critical for in vitro LipidII transport and in vivo FtsW function [34].

MurJ was identified in a bioinformatics search for possible LipidII flippases as an essential *Escherichia coli* gene coding for an IMP that is a member of the multidrug/oligosaccharidyl-lipid/polysaccharide (MOP) exporter superfamily, which includes transporters of other undecaprenyl-linked molecules. MurJ is conserved amongst PG containing bacteria but absent from bacteria that do not have PG [35]. Cells depleted for *murJ* show severe shape defects and have reduced incorporation of new PG material [35–37]. MurJ contains 14 TMS and its structure has been modeled, revealing a hydrophilic cavity containing charged residues that are essential for MurJ function [38,39]. The Ruiz and Bernhardt labs developed an in vivo flippase assay in which radiolabeled LipidII is cleaved after translocation by externally added ColM (a toxin that cleaves LipidII that is exposed in the periplasm). Both the ColM cleavage product and membrane associated LipidII can be detected, and the rationale of the assay is that a block of LipidII translocation results in a decrease of ColM cleavage product with a concomitant increase of LipidII in the membrane. Cells that expressed MurJ displayed LipidII transport activity, and, importantly, cells expressing a MurJ variant that could be chemically inactivated were blocked in LipidII translocation when the inactivator was added [40]. In this assay, LipidII translocation also occurred when the SEDS proteins RodA and FtsW were absent from the cell [40].

An exciting recent paper indicates that there is at least a third class of LipidII flippases. *Bacillus subtilis* is perfectly viable without its four most obvious MurJ paralogs [41], but even a strain lacking all ten MOP family members is viable [42]. This MOP-less strain allowed Rudner and colleagues to identify Amj (alternate to MurJ) as a novel type of LipidII translocase. Amj is synthetically lethal with *ytgP*, the MOP identified as the major *B*. *subtilis murJ* (MurJ_Bsu_) homolog, indicating that these genes have redundant functions [42]. Amj and MurJ_Bsu_ are both capable of rescuing an *E*. *coli murJ* deletion, and Amj functions in the ColM-LipidII flipping assay [42]. Amj is predicted to have six TMS and is neither a MOP family member nor an ATP-Binding Cassette (ABC) transporter. Amj is not widely conserved, yet it is present in subsets of both gram-positive and -negative bacteria.

It is difficult to reconcile the in vitro and in vivo flippase assays’ results. Each assay shows activity only for either FtsW and RodA or for MurJ and Amj (not tested in vitro). Inactivation of either FtsW or MurJ through a point mutation or a chemical modification abolishes its activity in the respective assay. From a biochemist’s perspective, the in vitro translocation assay [33] using proteoliposomes which contain only FtsW or MurJ is very clean, but MurJ may have inadvertently been inactivated during purification and reconstitution, precluding the measurement of MurJ activity. The in vivo assay [40] is equally elegant, but as it uses whole cells (or spheroplasts), it also leaves room for other explanations of the observations. What if LipidII uses both protein classes for transport, with different kinetics? The ColM assay requires LipidII to be available for ColM—if FtsW and/or RodA quickly hands over LipidII to its cognate transpeptidase for incorporation into PG, this might preclude ColM cleavage. MurJ-translocated LipidII might be more exposed in the outer leaflet of the membrane, thus allowing ColM to cleave only the MurJ-associated fraction of LipidII.

The identification of three families of LipidII flippases over the past years indicates tremendous progress and provides new targets for antibacterials. The question of how LipidII is precisely translocated by these various flippases remains to be resolved.

## LipidII and Membrane Organization

**Fig 2:**
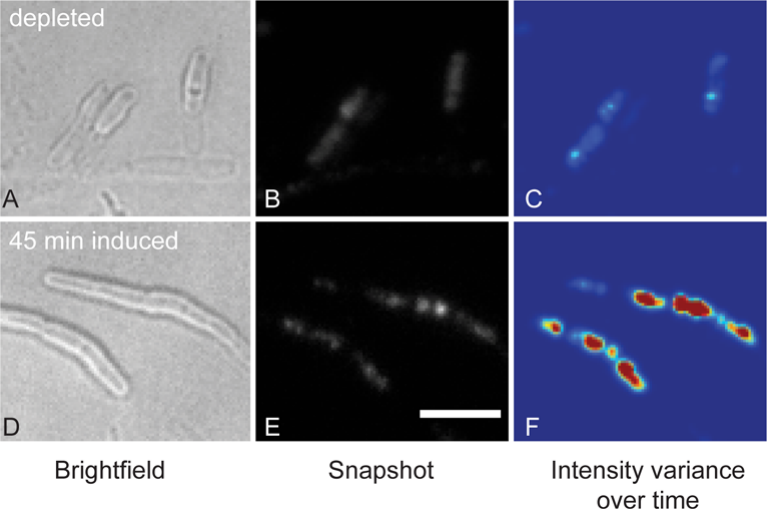
LipidII regulates membrane association of MreB. Using Total Internal Reflection Fluorescence (TIRF) microscopy, association and dissociation of MreB-GFP with the membrane can be followed. Upon depletion of MurG and subsequent halt of conversion of LipidI to LipidII, MreB-GFP is released from the membrane (upper row); after induction of MurG expression, LipidII production is resumed and MreB-GFP is re-localized on the membrane (lower row). Shown are snapshots (A, B, D, E) of single TIRF images at the respective time points and an analysis of the variance in intensity over time (C, F), with red indicating regions of high protein mobility and blue denoting low mobility. (Adapted with permission from Macmillan Publishers Ltd.: Nature Chemical Biology; K. Schirner et al., *Nat Chem Biol* 11, 38–45 [2015], Macmillan Publishers Ltd. 2015.)

LipidII binding by antibacterial compounds and its incorporation into PG take place at the outer leaflet of the cytoplasmic membrane. A possible role for LipidII in the inner leaflet of the membrane has not received much attention. This may change rapidly, as a recent study showed that LipidII regulates membrane association of the actin homologue MreB in *B*. *subtilis* [43]. Membrane association of MreB was known to be dependent on active PG synthesis [44,45], but the novel study suggests that it is the lack of carrier Lipid molecules that leads to MreB dissociation, not the absence of PG synthesis per se. Blocking synthesis of wall teichoic acid (WTA), depletion of UppS (required for undecaprenyl-pyrophosphate synthesis), and inhibiting PG synthesis all led to MreB release from the membrane [43]. Depletion of MurG, which blocks synthesis of LipidII but not of its precursor LipidI, also resulted in MreB delocalization, suggesting that MreB binds directly to LipidII (Fig 2) [43]. It has to be noted that many of these treatments alter the flux of substrates through the PG synthesis pathway [46], which could influence MreB dynamics [44,45] or the localization of other proteins that interact with MreB. In the case of MurG depletion, overall lipid organization in the membrane is disrupted [47], which may also cause MreB release.

**Fig 3:**
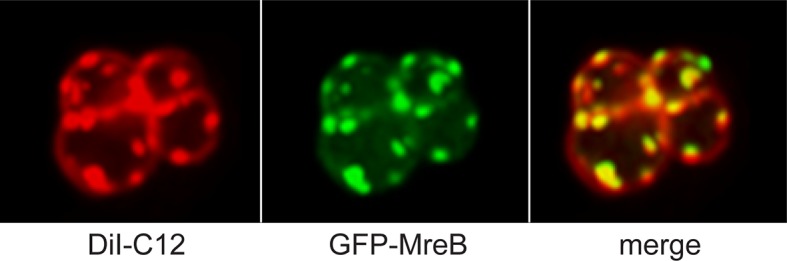
MreB is required for the generation of regions of increased fluidity (RIFs). GFP-MreB (green; panel B, C) co-localizes with regions of increased fluidity (RIFs, stained with the lipid-dye DiI-C12, red; panel A, C) in a ΔMreBCD strain of *B*. *subtilis* (cells look round because of the resulting shape defect). (Adapted with permission from H. Strahl, F. Burmann, L. W. Hamoen, The actin homologue MreB organizes the bacterial cell membrane. *Nat Commun* **5**, 3442 [2014].)

MreB is required for the generation of fluid lipid membrane domains, so-called regions of increased fluidity (RIFs) (Fig 3) [48]. LipidII partitions in the more mobile domains of supported bilayers that contain liquid-crystalline lipid domains segregated from more gel-like lipids [49], thus the presence of increased LipidII in these RIFs makes sense. When MreB and its homologues Mbl and MreBH are absent, RIFs disappear, and the membrane becomes more homogeneous—this also affects the diffusion of other membrane proteins [48]. Notably, RIFs are different from bacterial lipid raft domains [50,51], which are less fluid than the surrounding material [52].

**Fig 4:**
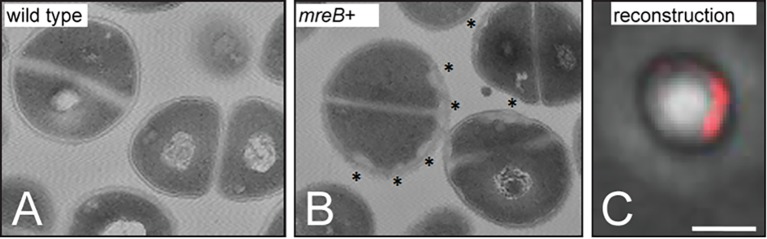
Expression of MreB in the non-MreB–containing bacterium *S*. *aureus* leads to accumulation of MreB and peptidoglycan. Patches of MreB are formed (red, panel C), leading to aberrant production of peptidoglycan (asterisks, panel B). Wild type cells are shown for comparison (panel A). (Amended with permission from American Society for Microbiology from A. Yepes et al., 2014, *Appl Environ Microbiol* 80, 3868–3878, DOI: 10.1128/AEM.00759-14.)

These two reports raise a paradox: does the presence of LipidII recruit MreB, or does MreB organize the membrane so that LipidII is recruited to domains to facilitate active PG synthesis—or is it a bit of both? Bacteria that lack MreB, such as *Staphylococcus aureus* and *Corynebacterium glutamicum*, do not organize the membrane in RIFs [48], yet do localize LipidII at sites of active PG synthesis [53]. MreB, heterologously expressed in *S*. *aureus*, forms patches at discrete regions in the membrane (not at the septum, the normal location of LipidII), and these MreB patches organize new call wall synthesis, resulting in misshapen cells (Fig 4) [54]. This experiment supports the notion that MreB recruits LipidII, either directly or indirectly. MreB localization does not solely depend on LipidII but also on various membrane proteins such as MreD [55,56] and RodZ [57–59]; MreB interacts with key proteins involved in LipidII synthesis [56], suggesting that a key function of MreB is to coordinate LipidII synthesis at regions where the membrane environment is “friendly” for LipidII. The presence of sufficient LipidII at these sites could act as a feedback mechanism that allows MreB dynamics and formation of a protein complex that drives PG synthesis along the lateral wall [43], and depletion of the LipidII pool in the membrane would result in a (temporary) halt of PG synthesis until order is restored [44,45,60].

Both studies on MreB recruitment by LipidII [43] and MreB-mediated membrane organization [48] were performed in *B*. *subtilis*. Membrane attachment of MreB in gram-positive bacteria is mediated by an internal hydrophobic loop, whereas gram-negative MreB contain an additional N-terminal amphipathic helix that is required for membrane binding [61]. This may result in a different affinity for the membrane; however, both attachment methods allow the binding of MreBs to synthetic vesicles devoid of or containing a very low amount of (in the case of total lipid mixtures) LipidII [61–63]. Such in vitro methods, in which liposomes can be doped with synthetic LipidII, can be used to further study the role of LipidII in MreB recruitment, the role of MreB in the organization of membrane domains, and the presence of LipidII in such domains.

## Organization of PG Synthesis

**Fig 5:**
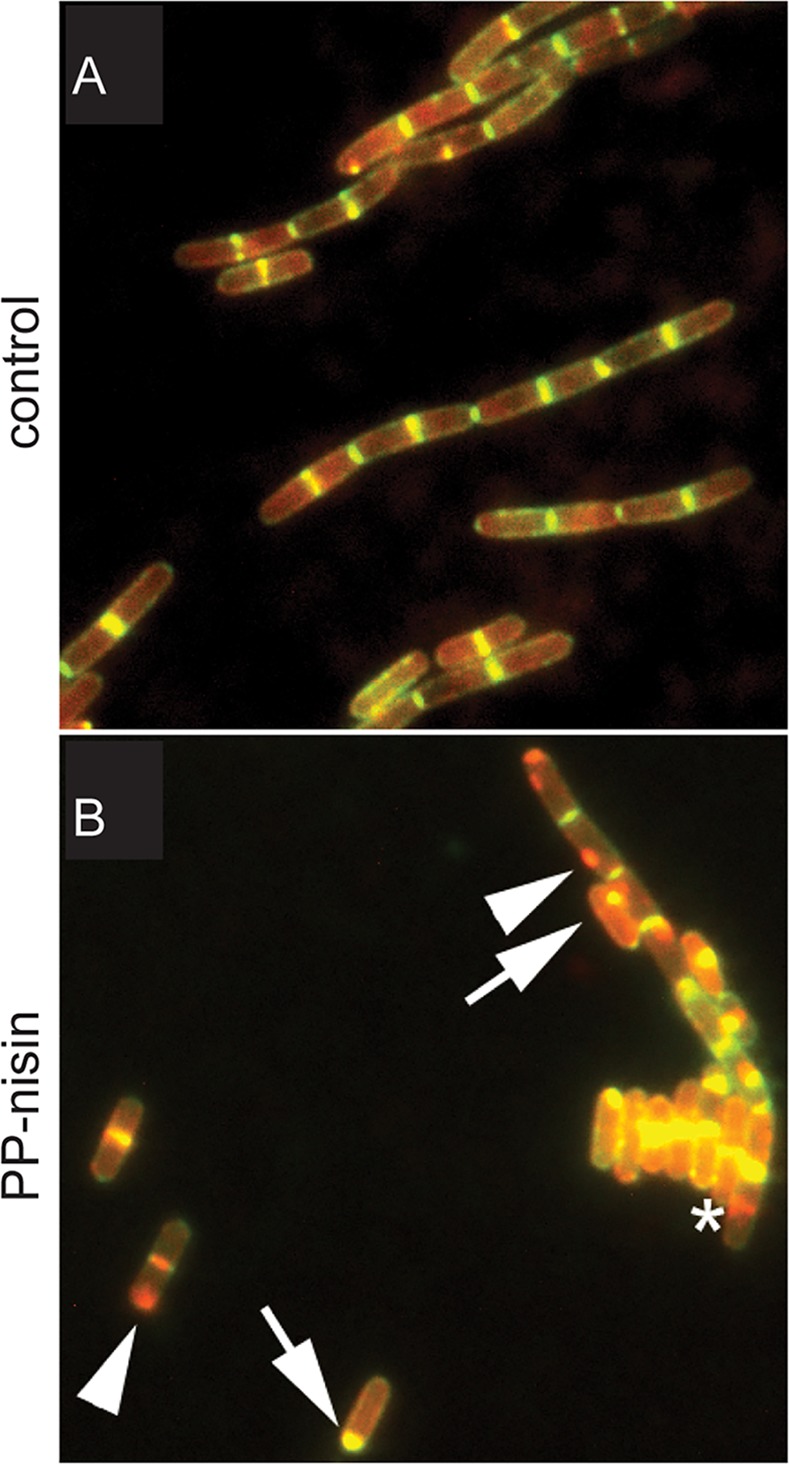
Clustering of LipidII in nonphysiological domains leads to recruitment of elongation-specific PBPs. Under normal circumstances, LipidII (stained with fluorescent vancomycin [Van-FL], green) and RFP-PBP2A (red) co-localize at the septum (yellow in control, panel A) and the lateral wall. When LipidII is clustered into nonphysiological domains with PP-nisin, RFP-PBP2A follows LipidII in 94% of the cases when cells exhibit both LipidII and PBP2A spots (panel B, arrows, strong co-localization; arrowheads, co-localization but weak Van-FL signal). (Adapted from The localization of key Bacillus subtilis penicillin binding proteins during cell growth is determined by substrate availability, Lages MC, Beilharz K, Morales Angeles D, Veening JW, Scheffers DJ, *Environmental Microbiology* 15, 3272–3281 [2013], John Wiley & Sons, Inc. http://onlinelibrary.wiley.com/doi/10.1111/1462-2920.12206/abstract.)

LipidII incorporation into PG occurs through a combination of transglycosylation reactions that attach the disaccharide to glycan strands and transpeptidation reactions that form crosslinks between the pentapeptide chains that are attached to the glycan strands. These reactions are mediated by the so-called penicillin-binding proteins (PBPs), the targets for beta-lactam antibiotics. The PBPs are organized in large protein complexes that span the cytoplasmic membrane and that include proteins involved in LipidII synthesis, translocation, and incorporation into PG—known as the “divisome” for synthesis of the division septum and “elongasome” for synthesis of lateral cell wall in non-coccoid bacteria [4,64]. The divisome and elongasome are organized by the cytoskeletal proteins FtsZ and MreB, respectively. Some bacteria, like *Chlamydia sp*., synthesize PG without apparent coordination of cytoskeletal elements [9,10]. But in organisms that contain cytoskeletal elements, the presence of LipidII plays an important role in localizing the PG synthesis machinery through a process called substrate availability [65]. Altering LipidII structure, or blocking LipidII binding by PBPs by either vancomycin or β-lactams, leads to delocalization of the critical PBP2 in *S*. *aureus* [66]. In *Streptococcus pneumoniae*, the localization of several PBPs to the zone of PG synthesis is controlled by PBP3, a carboxypeptidase that cleaves the terminal D-Ala from pentapeptide chains [67]. In the absence of PBP3, pentapeptide substrates accumulate over the cell surface, causing several PBPs to delocalize to these zones of potential PG synthesis [67,68]. The localization of PBP3 was reported to be either on the whole cell surface, including the division site [68], or only on the cell surface but occluded from the division site [67]. Although the localization of PBP3 is not resolved, it is clear that PBP3 controls the availability of PG precursors and thus localization of PG synthesis. *S*. *aureus* and *S*. *pneumoniae* do not contain MreB, but in organisms where MreB is present, substrate availability is also important, strongly suggesting that PBPs are not just tethered to the PG synthesis complex through FtsZ and/or MreB, but in addition require the presence of (and the capability to bind to) substrate to localize at the right site. The *E*. *coli* carboxypeptidase PBP5 delocalizes from the division site, where it is most active, when its active site is mutated, and it accumulates even more at the division site when cell wall synthesis along the lateral wall is inhibited [69]. In *Caulobacter crescentus*, PBP3 delocalizes when its active site is mutated [70]. Key components of the *B*. *subtilis* elongasome, PBP2A and PbpH, are recruited to clusters of LipidII when LipidII is actively clustered in nonphysiological domains in the membrane (Fig 5) [71]. The recruitment to LipidII patches was strictly dependent on LipidII, as PBP2A and PbpH did not delocalize in cells that were depleted for LipidII or when MreB was delocalized by a collapse of the membrane potential [71]. Combined, these studies provide a strong indication that the presence of LipidII functions as a targeting signal for peptidoglycan synthesis proteins.

## Concluding Remarks

Despite the great progress made in the last years, many questions on LipidII biology remain. The identification of various LipidII flippases opens up a new field of study and will hopefully lead to the elucidation of the molecular mechanism(s) of LipidII flipping. The proposed roles of LipidII in organizing cytoskeletal proteins and peptidoglycan synthesis complexes on both sides of the membrane require a combination of biochemical and cell biology approaches to fully understand to which degree LipidII plays an active role in this organization, or whether LipidII just happens to be at the right place at the right time. Finally, the discovery of teixobactin has again underscored the importance of LipidII as a target for antibacterials that are not (very) susceptible to rapid resistance development. Hopefully, more LipidII-targeting compounds with promise for clinical use will be discovered in the near future.
